# Mechanical Performance and Constitutive Model Analysis of Concrete Using PE Fiber-Strengthened Recycled Coarse Aggregate

**DOI:** 10.3390/polym14193964

**Published:** 2022-09-22

**Authors:** Qimi Zhou, Yingwu Zhou, Zhipei Guan, Feng Xing, Menghuan Guo, Biao Hu

**Affiliations:** 1 College of Civil and Transportation Engineering, Shenzhen University, Shenzhen 518060, China; 2 Guangdong Provincial Key Laboratory of Durability for Marine Civil Engineering, Shenzhen University, Shenzhen 518060, China; 3 Key Laboratory for Resilient Infrastructures of Coastal Cities, Ministry of Education, Shenzhen University, Shenzhen 518060, China

**Keywords:** recycled coarse aggregate (RCA), limestone calcined clay cement (LC^3^), seawater and sea sand (SWSS), strength, stress-strain relationship, constitutive model

## Abstract

To promote the sustainable development of the construction industry, concrete incorporating polyethylene (PE) fiber-strengthened recycled coarse aggregate (SRCA) and seawater and sea sand (SWSS) is prepared. The usage of SRCA significantly improves the mechanical performance of concrete. The strength is improved, and the failure mode of concrete cylinders is also remarkably altered. The incorporation of SWSS that alleviates the shortage of freshwater and river sand slightly reduces the mechanical strength of concrete at 28 and 90 days, while the replacement of cement by 35% limestone calcined clay cement (LC^3^) overcomes this drawback. The compressive strength of concrete is further enhanced, and the pore structure is refined. The introduction of LC^3^ also promotes the formation of Friedel’s salt, which could improve the chloride binding capacity of concrete using SWSS. Furthermore, the stress-strain relationship of sustainable concrete is analyzed, and the experimental results are compared with the commonly used constitutive models. The predictive constitutive models are proposed to effectively describe the mechanical performance of sustainable concrete.

## 1. Introduction

With the rapid development of urbanization, the process of urban construction renewal is accelerating. On the one hand, natural coarse aggregates are excessively consumed. on the other hand, a large amount of construction and demolition (C&D) waste is generated. The rampant stacking as well as the landfilling of a huge amount of C&D waste not only causes environmental pressure, but also occupies land resources. From the perspective of sustainable development, the recycling of waste concrete is an effective way to solve the above mentioned problems [1,2,3,4]. Extensive research has been carried out concerning the mechanical properties of recycled concrete (RC) [5,6,7,8,9,10,11,12,13]. However, as a certain amount of old mortar is attached in recycled coarse aggregate (RCA), RCA exhibits intrinsic defects, such as high porosity, high water absorption ratio and low mechanical resistance [14]. Moreover, micro-cracks pre-exist in RCA due to the preparation process of crushing and grinding [2]. To improve the performance of RCA, various treatment methods are proposed by researchers worldwide [15]. Kou et al. [16] and Zhan et al. [17] have used the carbonization method to densify RCA by converting Ca(OH)_2_ in old mortar to CaCO_3_. Mukharjee et al. [18] have incorporated 3% nano-silica (NS) in RC, and the increasing ratio of compressive strength was found to be 18%. Other researchers have also used NS to improve the microstructure as well as the macroscopic mechanical properties of RC [19,20,21]. The microbial carbonate precipitation method was also suggested by Qiu et al. [22] to enhance the properties of RCA. The additionally formed calcium carbonate filled the pores in RCA, and thus densified the microstructure of aggregates. In addition, strengthening RCA with a layer of cementitious material is also investigated by some researchers to enhance RCA [23]. The hydration products of the strengthening layer could fill the pores and microcracks in RCA and thus improve the quality of aggregate [23]. This strengthening methodology is employed in this work, and polyethylene (PE) fiber reinforced cementitious material was used to prepare the strengthened recycled coarse aggregate (SRCA).

Many countries and regions today are facing fresh water shortages, and by 2025, 5.5 billion people in the world will be under water pressure [24]. The world produces 30 billion tons of concrete every year, while the consumption of fresh water reaches 2 billion tons [25]. At the same time, river sand, as one of the main components of concrete, is also facing the problem of resource shortage. River sand reserves have rapidly declined in some areas as a result of long-term extensive mining, and in some places, there is even no sand to mine. Excessive exploitation of river sand can seriously affect the normal use of river channels and the stability of riverbeds, and even threaten the structural safety of flood control dams in some areas, and if not controlled and prevented, it may cause significant human and property losses [26]. The use of abundant seawater and sea sand (SWSS) to make concrete not only alleviates the dilemma of resource shortage, but also greatly reduces transportation costs in coastal areas [14,27]. At present, a large number of researchers have carried out extensive research concerning the properties of seawater and sea sand concrete (SWSSC), and fruitful achievements have been achieved [14,28,29,30,31,32]. Since all the factors including water to binder (W/B) ratio, the salinity of seawater, the properties of sea sand and the addition of supplementary cementitious materials (SCMs) affect the properties of SWSSC [32], the reported findings sometimes are contradictory. Guo et al. [28] studied the effect of ion content in SWSS on the mechanical properties of concrete by selecting SWSS from different sea areas, and the results showed that SWSS would lead to a slight decrease in the compressive strength of concrete, but the overall mechanical properties of SWSSC were still comparable to ordinary concrete. Some studies have reported that SWSSC can obtain similar mechanical properties to ordinary concrete, and even higher compressive strength [29,30]. Other studies have reported that the introduction of SWSS reduces the compressive strength of concrete due to the presence of harmful ions and impurities in SWSS [27]. It is generally accepted that the addition of SWSS improves the early-age compressive strength of concrete owing to the accelerated hydration process by the chemical ions in SWSS, and that SWSSC can achieve comparable mechanical properties of ordinary concrete [28,33]. The key issue for the application of SWSS is to solve the corrosion problem of steel reinforcement caused by Cl^−^ in SWSS [34,35,36].

The usage of SCMs to replace one part of cement is an effective way to improve the chloride binding capacity of SWSS concrete [35,37]. The commonly used SCMs include fly ash or slag. However, the supply of fly ash and slag is limited, which cannot meet the huge demand for concrete production [37,38]. Clay containing kaolinite is abundant, and could be calcined to form the pozzolanic materials, i.e., metakaolin (MK). Using a combination of calcined clay and limestone to replace one part of cement clinker, the low carbon sustainable concrete could be achieved [37,38,39]. MK in calcined clay is composed of aluminates and could promote the formation of Friedel’s salt when meeting with chloride ions in SWSS [35,40]. The reported results show that limestone calcined clay cement (LC^3^) concrete exhibits comparable mechanical strength and even shows superior durability performance compared with ordinary concrete [39,41,42]. Antoni et al. [43] used LC^3^ with a total substitution ratio of 45% (a combination of 30% metakaolin and 15% limestone) to replace ordinary Portland cement (OPC), the 7d compressive strength of LC^3^ cement mortar was increased by 10%, the compressive strength of 28d was increased by 15%, and when LC^3^ with a total substitution ratio of 60%, its 28d compressive strength could also reach 93% of OPC. Dhandapani et al. [39] found that concrete with a total replacement ratio of 50% for LC^3^ had better compressive strength than OPC concrete at all the studied ages including 2, 7, 28, 90 and 365 days. It can be seen that LC^3^, a low carbon cementitious material, has obvious advantages for improving the properties of SWSSC.

In this paper, the sustainable concrete prepared with SRCA, SWSS and LC^3^ is investigated. The usage of untreated SWSS will facilitate the engineering construction in coastal area and remote island, and the sustainable concrete could be used in combination with corrosion resistant reinforcement. Thus, it is necessary to investigate the mechanical performance and the microstructural characteristics of this new type of concrete. The stress-strain behavior of concrete is discussed in detail, and the constitutive relationship is also investigated. Additionally, the predictive constitutive models are proposed by considering the influence of concrete age.

## 2. Preparation Process of Strengthened Recycled Coarse Aggregate

**Figure 1:**
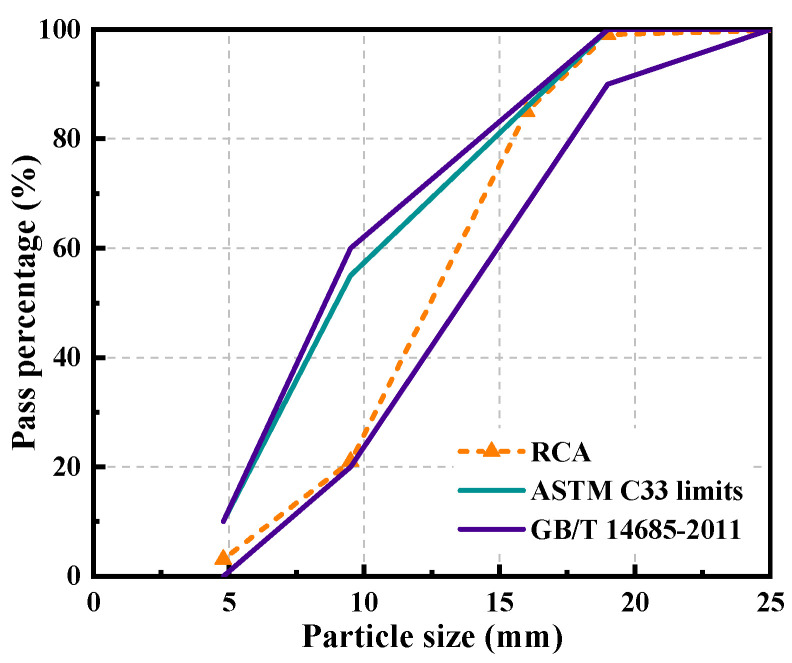
Particle size distribution of RCA.

**Figure 2:**
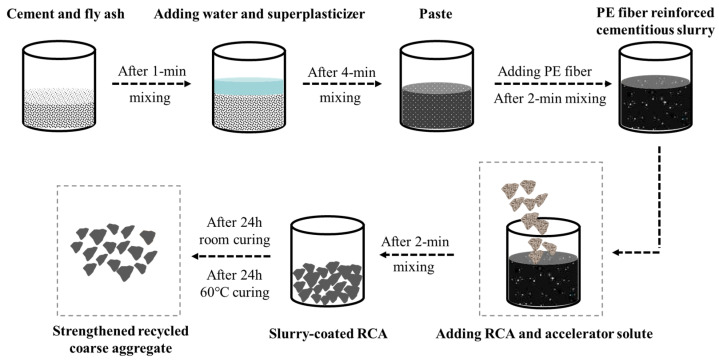
Preparation process of SRCA.

**Figure 3:**
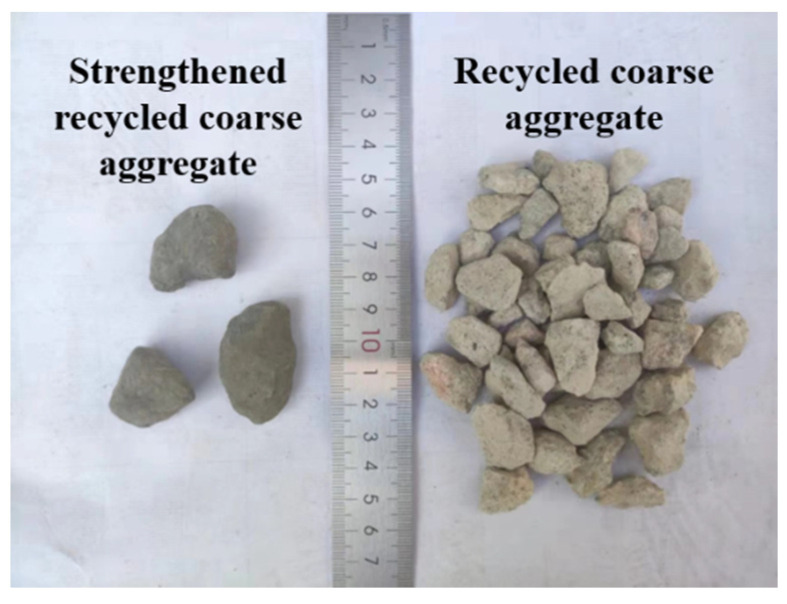
Comparison of SRCA and RCA.

RCA was obtained by crushing unknown waste concrete collected from a local C&D waste site in Shenzhen, China. The particle size range of RCA is 5~20 mm, as shown in Figure 1. The bulk density, crushing index, and water absorption of RCA are summarized in Table 1. Strengthened recycled coarse aggregate (SRCA) was prepared by wrapping RCA with a layer of PE fiber-reinforced cementitious slurry. The mixture proportion of the strengthening slurry is summarized in Table 2. Firstly, the cementitious slurry was prepared by mixing cement, fly ash, water and superplasticizer. Then, PE fiber with the properties detailed in Table 3 was added in the mixture. After proper dispersion of fibers, RCA and accelerator solute were placed into the mixer. Once RCAs were completely covered by the fresh slurry, they were pulled out. After air drying for 24 h, SRCA were placed in a 60 °C drying oven for 24 h. The flow chart of the preparation process is illustrated in Figure 2. The obtained SRCAs are shown in Figure 3.

After strengthening, the crushing index of SRCA is reduced to be 12.21% with the reduction ratio being 37.35%; the water absorption ratio of SRCA being 6.02% kept almost unchanged, as shown in Table 1. The obvious reduction of the crushing index indicates the mechanical resistance of SRCA is significantly increased, which implies that the strengthening method could effectively improve the properties of RCA.

## 3. Experimental Program

### 3.1. Materials

**Figure 4:**
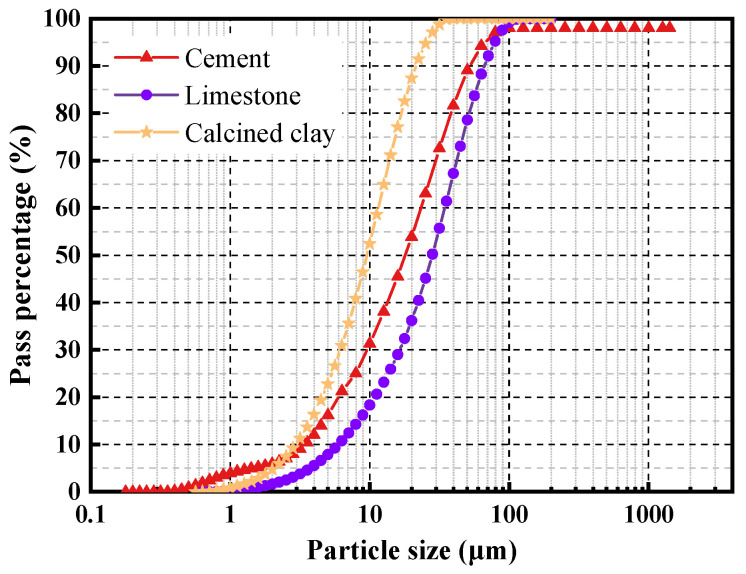
Particle size distribution of the cementitious materials.

The cement used is OPC 42.5R. LC^3^ is composed of OPC, calcined clay and limestone. The chemical composition and physical properties of the cementitious materials is presented in Table 4, and the particle size distribution is shown in Figure 4. The Bogue composition of OPC is 57.26% C_3_S, 15.08% C_2_S, 9.31% C_3_A, and 12.46% C_4_AF. Two replacement ratios of OPC by LC^3^, 35% and 50%, were investigated. Fine aggregates used in this work are sea sand and desalted sea sand. The detailed properties of fine aggregates are illustrated in Table 5. The studied water types include tap water and seawater, and the main ion contents of the two kinds of water are demonstrated in Table 6.

### 3.2. Mixture Proportions of Concrete

In order to evaluate the influence of the strengthening method of RCA on the properties of concrete, RC, the reference concrete prepared with untreated RCA, tap water and desalted sea sand, and SRC, the concrete made with SRCA, tap water and desalted sea sand were investigated. To characterize the effect of seawater and sea sand on the properties of concrete, SSRC, the concrete prepared with SRCA, seawater and sea sand was studied. To explore the influence of LC^3^ on the performance of concrete, L(35) SSRC, representing the concrete prepared with SRCA, seawater, sea sand and 35% substitution ratio of OPC by LC^3^, and L(50) SSRC, representing the concrete prepared with SRCA, seawater, sea sand and 50% substitution ratio of OPC by LC^3^ were investigated. The mixture proportions of the five concrete types are illustrated in Table 7. Additional water was added to compensate the absorbed water by RCA and SRCA, and the content of additional water was calculated by considering that the recycled aggregate attaining saturated surface-dried state. Thus, the effective water/binder (w/b) ratio of each mixture is 0.39. The slump values of the studied concrete types are about 50 mm, while the air content and concrete temperature were not tested.

### 3.3. Compression Test

Cylindrical specimen with a diameter of 150 mm and a height of 300 mm was used for the axial compression test. Two curing ages including 28 days and 90 days were considered. The 3000 kN MTS-YAW6306 pressure testing machine (MTS Systems (China), Shenzhen, China) was applied for loading. The displacement-controlled load was applied at the rate of 0.3 mm/min until the failure of the specimen. The linear variable displacement transducers (LVDTs) were used to measure the axial displacement of the specimen. The Dewesoft dynamic collection box (Dewesoft (Beijing, China) Measurement and Control Technology, Beijing, China) was used to collect the load and displacement data during the loading process.

### 3.4. MIP Test

To explore the influence of LC^3^ and SWSS on the microstructural characteristics of concrete using SRCA, mercury intrusion porosimetry (MIP) test was performed. The samples were cut off from the ruptured concrete, and the coarse aggregates were eliminated. The hydration process was terminated by immersing the specimens in absolute ethyl alcohol. Then, the specimens were vacuum dried at 60 °C for 24 h before testing. Poromaster GT-60 Mercury Intrusion Porosimeter, Quantachromre (Boynton Beach, FL, USA), was used for MIP testing.

### 3.5. XRD Test

To further investigate the influence of LC^3^ and SWSS on the microstructure of concrete using SRCA, X-ray diffraction (XRD) analysis was performed to identify the hydration phase assemblages of the four paste types of SRC, SSRC, L(35)SSRC and L(50)SSRC. The pastes were prepared in parallel with the concrete cylinders and cured to 28 days. After the termination of hydration process, the paste was vacuum dried at 60 °C for 24 h. Then, the paste samples were ground into powder before XRD testing. During the test, Cu Kα radiation (λ = 1.54 Å) at 40 kV and 40 mA was applied, and the scanning was performed at a speed of 0.02°/step from 5° to 65°.

## 4. Mechanical Properties of Concrete

### 4.1. Stress-Strain Relationship

**Figure 5:**
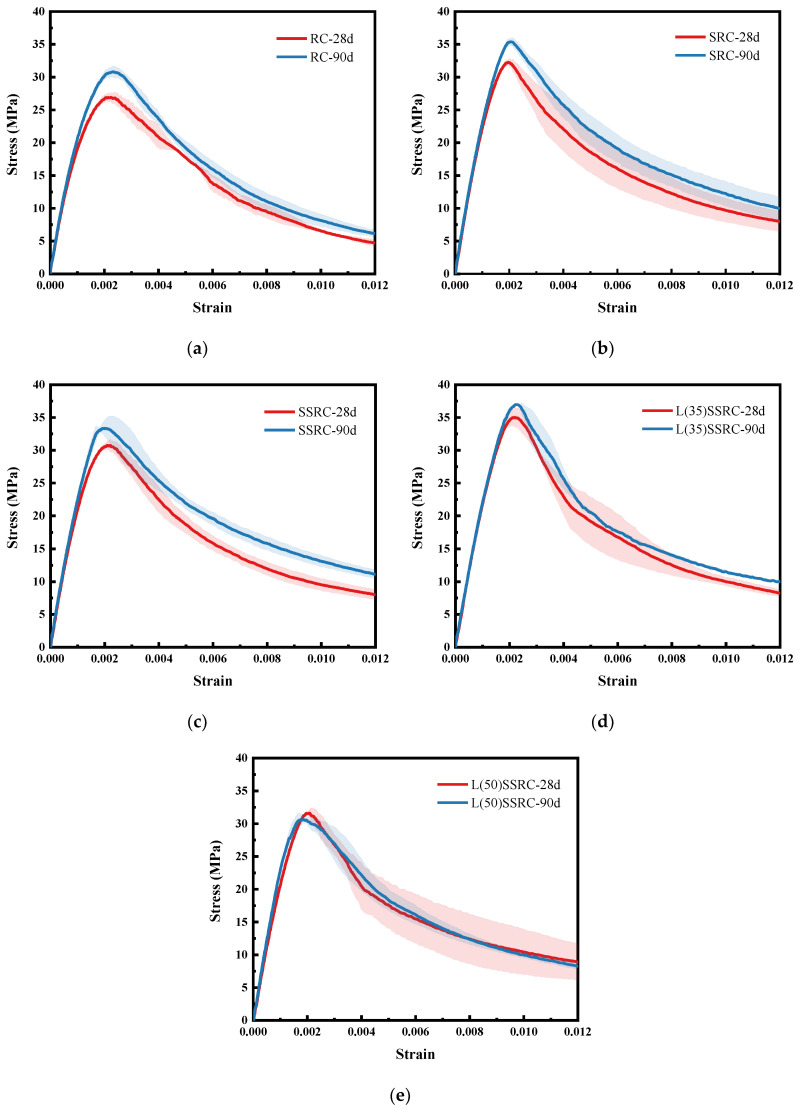
Average stress-strain curves of the five types of concrete at 28 and 90 days (**a**) RC, (**b**) SRC, (**c**) SSRC, (**d**) L(35)SSRC, and (**e**) L(50)SSRC.

**Figure 6:**
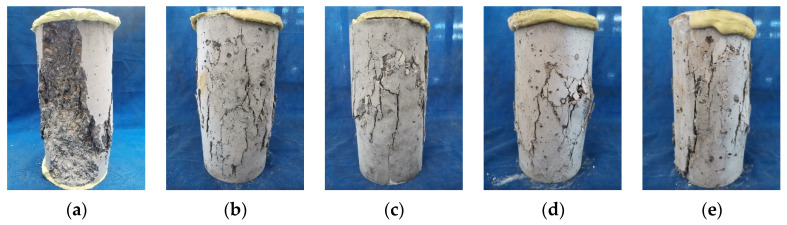
Failure modes of concrete (**a**) RC, (**b**) SRC, (**c**) SSRC, (**d**) L(35)SSRC, and (**e**) L(50)SSRC.

The average stress-strain curves of various concrete cylinders at the age of 28 and 90 days are shown in Figure 5, and the data distribution range is also marked. All the stress-strain curves exhibit three development stages, i.e., linear increasing stage, non-linear rising stage, and descending stage. It is remarked that the mechanical performance of concrete using SRCA is remarkably improved at both 28 and 90 days by comparison with the concrete using untreated RCA. The stress-strain curve of SRC almost covers that of RC, with the strength increasing ratio being 19.34% at 28 days and 15.13% at 90 days. According to the failure modes of concrete, demonstrated in Figure 6, the concrete blocks are peeled off from the cylinders of RC concrete, and the damage occurred in recycled aggregates with the ruptured surface of aggregates exposed in the failure cross section. In contrast, the cylinders of SRC concrete keep almost intact with only cracks distributed in the specimen. No obvious peeling concrete blocks are observed. The fiber reinforced strengthening layer impedes the propagation of main cracks and contributes to the development of distributed cracks. The energy dissipation capacity of SRC concrete is improved, and its failure mode is totally changed.

When seawater and sea sand are introduced in concrete, the mechanical behavior is slightly deteriorated. The compressive strengths of SSRC are slightly lower than those of SRC at both 28 and 90 days. The failure mode of SSRC is similar to that of SRC, as shown in Figure 6. Once 35% OPC is replaced by LC^3^, the strength of concrete L(35)SSRC is further improved at the two studied ages, as shown in Figure 5. The stress-strain curve of L(35)SSRC covers all the other curves at 28 days, and the failure mode of L(35)SSRC is similar to that of SSRC. When 50% LC^3^ is incorporated in concrete, the mechanical performance is deteriorated. The strength reduction ratios of L(50)SSRC attain 10.56% at 28 days and 16.24% at 90 days compared to L(35)SSRC. The failure mode of L(50)SSRC is also altered with the main splitting cracks formed. However, the cylinder still keeps complete with few blocks peeling off from the specimen. It could be concluded that the fiber reinforced strengthening layer limits the interlinking of cracks, and thus the falling off of broken fragments is avoided, regardless of cement types, water types and fine aggregate types.

### 4.2. Peak Stress

**Figure 7:**
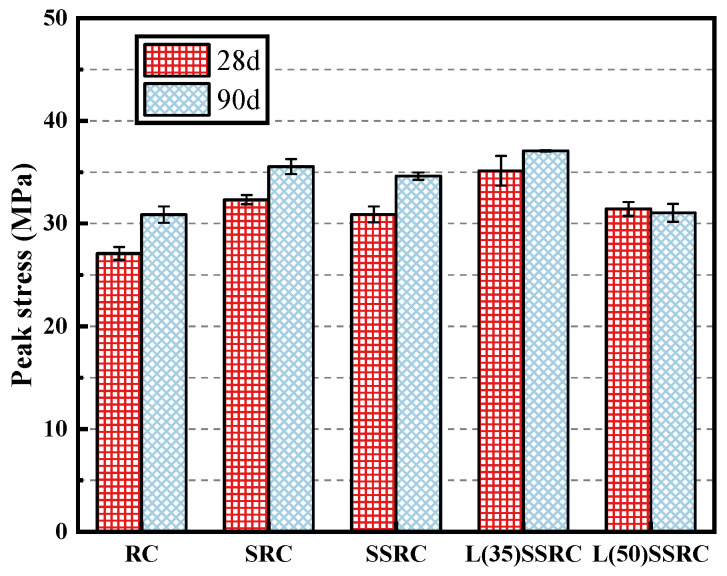
Peak stress of concrete cylinders at 28 and 90 days.

Figure 7 demonstrates the development of peak stress of the five studied concrete types. As mentioned in the previous section, the usage of SRCA improves the mechanical resistance of concrete. The fiber reinforced cementitious layer could enhance the properties of old adhered mortar, and thus strengthens the interfacial transition zones (ITZ) between recycled aggregate and new mortar matrix. The embedded fibers could run through the interphase between aggregate and mortar, and improves the load transfer capacity of ITZ, which also benefits impeding the propagation of main cracks. The aggregate type plays a crucial role in determining the strength of recycled concrete. When seawater and sea sand are incorporated, the various chemical ions promote the early-age hydration of cement [32,44,45] and increase the early strength of concrete. However, the late strength growth is limited due to the formation of porous microstructures [28]. The peak stress of SSRC is slightly reduced compared to SRC at 28 and 90 days. Nevertheless, the peak stress of concrete using seawater and sea sand could be enhanced by the incorporation of LC^3^. The reactive silica and alumina in calcined clay could react with the hydration product Ca(OH)_2_, and lead to the formation of C-A-S-H [46]. The aluminates in both calcined clay and cement could react with CaCO_3_ from limestone to form mono- (Mc) and hemi-carboaluminates (Hc), and the carboaluminates transfer to Friedel’s salt when chloride ion exists in the hydration system [47]. This phenomenon is analyzed in detail in Section 5.2. The newly formed hydration phases fill in the micropores and densify the microstructure of L(35)SSRC. However, the improving effect of LC^3^ is impaired when the replacement ratio rising to 50%. The effective water/binder ratio is significantly reduced when 50% LC^3^ is used, and the decrease of clinker content lowers the build-up of hydration phases. Especially, the content of available Ca(OH)_2_ is reduced, and thus the pozzolanic reaction between calcined clay and Ca(OH)_2_ is limited. This phenomenon results in the adverse impact on the strength of concrete. According to Krishnan [48], when the replacement ratio of LC^3^ is 55%, only a small fraction (45.6%) of MK is reacted, and about 50% of MK is not involved in the hydration process.

### 4.3. Elastic Modulus and Peak Strain

**Figure 8:**
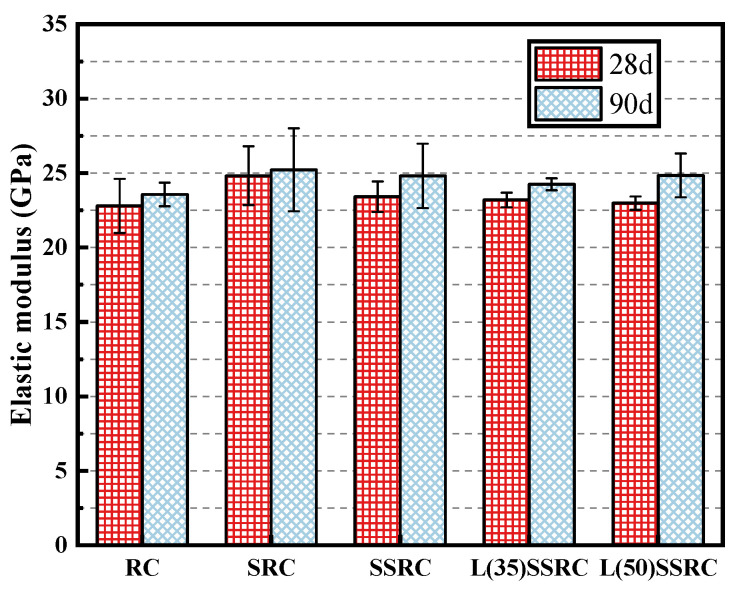
Elastic modulus of concrete at 28 and 90 days.

**Figure 9:**
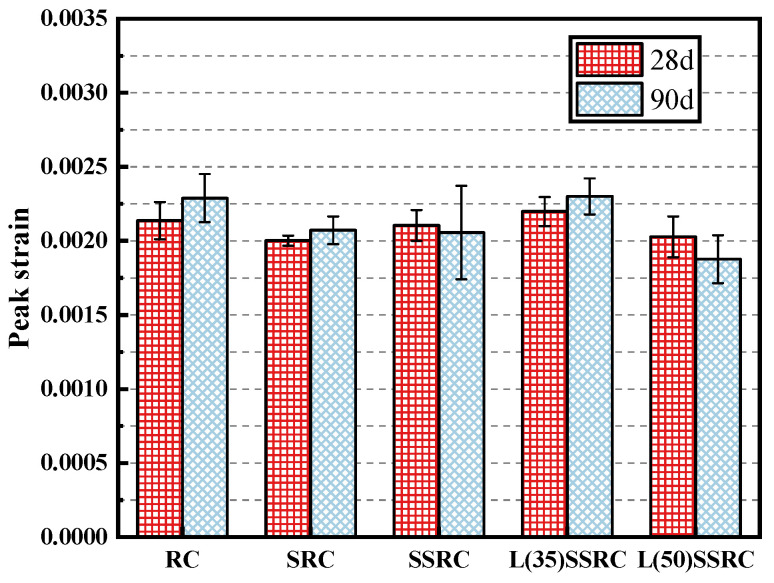
Peak strain of concrete at 28 and 90 days.

The elastic modulus of each concrete type was calculated according to GB/T 50081-2002 “Standard for Test Methods of Mechanical Properties of Ordinary Concrete” [49], that is, the secant slope of 1/3 peak stress and 0.5 MPa in the stress-strain curve. The results are presented in Figure 8. The elastic modulus of concrete using SRCA is increased by 8.9% and 7.0% at 28 and 90 days, respectively, compared to RC. The introduction of seawater and sea sand slightly decreases the elastic modulus of concrete. The incorporation of LC^3^ also demonstrates slight influence on the value of elastic modulus. However, the elastic modulus of L(35)SSRC and L(50)SSRC are still comparable to those of SSRC. Furthermore, for each concrete type, the elastic modulus of concrete rises with the increase of curing age. As for the peak strain, the usage of SRCA slightly decreases the value, while the incorporation of seawater, sea sand and 35% LC^3^ further enhance the strain value (Figure 9). It could be concluded that 35% replacement ratio of OPC by LC^3^ benefits the achievement of both high strength and high strain of concrete using SRCA and SWSS.

## 5. Microstructure of Concrete

### 5.1. Pore Structure

**Figure 10:**
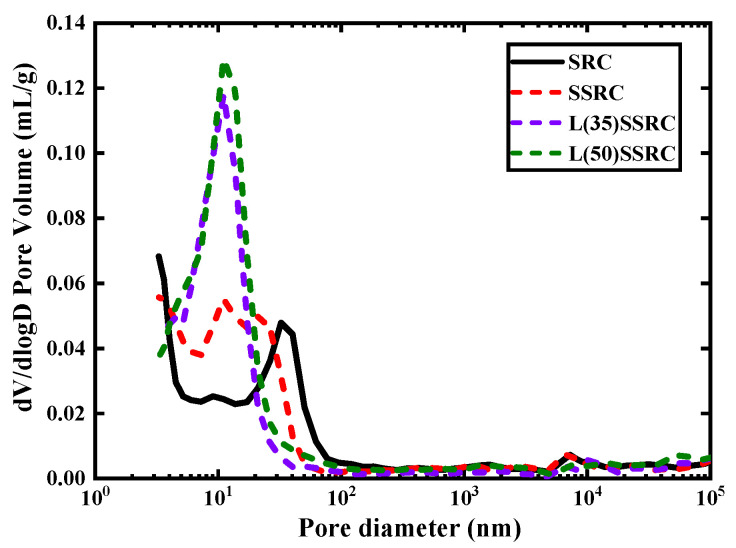
Pore size distribution of each studied concrete type.

**Figure 11:**
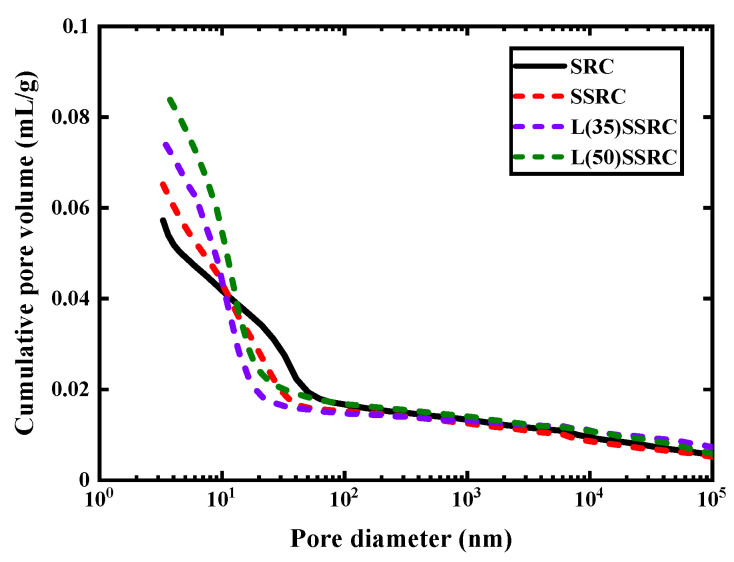
Cumulative pore volume of each studied concrete type.

**Figure 12:**
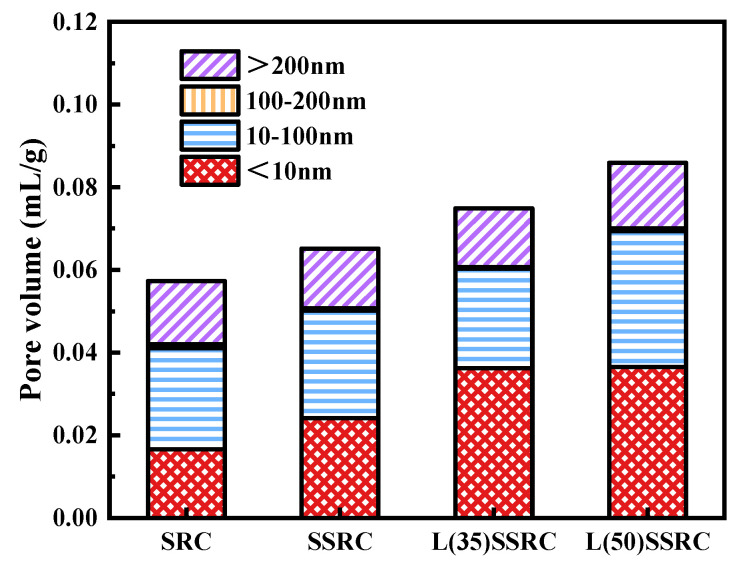
Pore distribution of different particle sizes of each studied concrete type.

The pore structural characteristics of studied concrete types are presented in Figure 10, Figure 11 and Figure 12. According to the pore size distribution curve, shown in Figure 10, the pores are refined by using SWSS, and the refinement is further significantly improved by incorporating LC^3^. Etxeberria et al. [50,51] have reported that the average pore size of concrete using SWSS, and recycled aggregate is reduced compared to recycled concrete using freshwater, which is probably originated from the salts existing in SWSS. The threshold pore size of SSRC matrix is reduced by comparison with that of SRC matrix, as shown in Figure 11. The reduction of the threshold pore size of L(35)SSRC and L(50)SSRC matrix is more significant. Although the total pore volume is increased with the incorporation of both SWSS and LC^3^, the increment is mainly attributed to the rising of the volume of pores smaller than 10 nm, i.e., gel pores [52]. The gel pores exhibit negligible influence on the strength of concrete [53]. Thus, the strength of concrete L(35)SSRC is enhanced compared to SSRC though the total porosity of L(35)SSRC is higher than that of SSRC. While for L(50)SSRC, the volume of pores larger than 10 nm, i.e., capillary pores, is increased compared to L(35)SSRC, the increment of capillary pores is detrimental to the strength of concrete. Correspondingly, the compressive strength of L(50)SSRC is lower than that of L(35)SSRC.

### 5.2. Hydration Products

**Figure 13:**
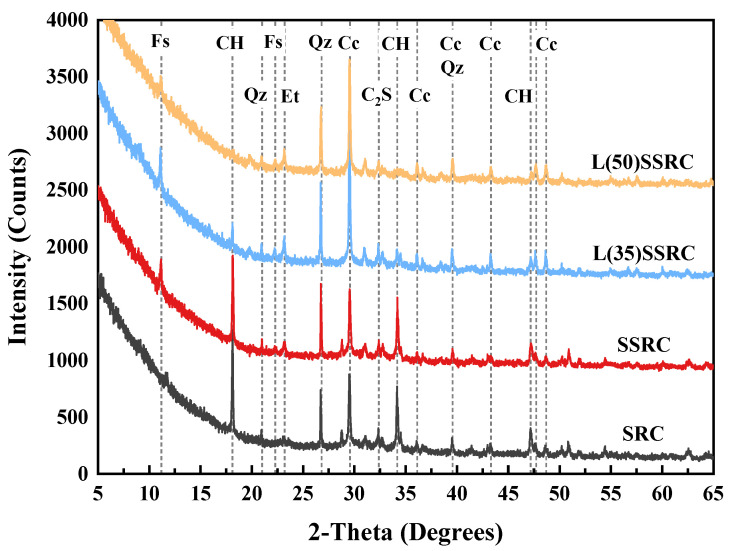
XRD patterns of concrete pastes. (Fs: Friedel’s salt; CH: Portlandite; Qz: Quartz; Et: Ettringite; Cc: Calcium carbonate; C_2_S: Dicalcium silicate).

The XRD patterns of the four studied paste types are demonstrated in Figure 13. Hydration products such as Friedel’s salt, calcium hydroxide, ettringite and calcium carbonate were identified in SSRC, L(35)SSRC and L(50)SSRC groups. Since Friedel’s salt is the typical hydration product generated by the reaction between chloride and aluminates in seawater cement pastes [33,54], this phase is not identified in the reference group SRC. The introduction of SWSS also promotes the formation of ettringite [55]. Several studies have shown that monocarboaluminate (Mc) and hemicarboaluminate (Hc) are formed in LC^3^ systems owing to the reaction between CaCO_3_ from limestone and the aluminates from calcined clay and cement [42,56,57,58,59]. However, Mc and Hc are not identified in the SWSS systems, the carboaluminates were converted to Friedel’s salts when meeting with chloride ions incorporated by SWSS [42,60]. Some researchers [27,40,54] have found that, in cement pastes mixed with MK and seawater, Mc and Hc were converted to Friedel’s salts and hydroaluminates (Ca_8_Al_4_(OH)_24_(CO_3_)Cl_2_(H_2_O)_1.6_(H_2_O)_8_), the latter of which is a solid solution between carboaluminates and Friedel’s salts. Additionally, calcium hydroxide (CH) content was significantly reduced due to the pozzolanic reaction with the reactive components in calcined clay [33,34,36]. The high content of aluminates in calcined clay promotes the formation of Friedel’s salt, which could fill the pores and is conducive to the densification of microstructure. Moreover, the content of calcium carbonate in L(35)SSRC and L(50)SSRC increases significantly owing to the incorporation of limestone. The calcium carbonate particles could act as filling materials [43] and decreases the interparticle spaces of the cementitious binder, which contributes to the densification of the microstructure of LC^3^ system. Furthermore, since the excess calcium carbonate present in the LC^3^ system prevents the converting of ettringite to monosulfate [41,56], the contents of ettringite in L(35)SSRC and L(50)SSRC are found to be higher than those in SRC and SSRC.

## 6. Constitutive Model Analysis

The stress-strain relationship of concrete is the basis of concrete structure design and is crucial for the application of concrete. In this study, the existing constitutive models were analyzed in combination with the experimental data, and the optimum constitutive models that fit well with the mechanical behavior of the studied concrete types were selected.

### 6.1. Existing Constitutive Models

Various researchers reported extensive experimental and theoretical studies on the constitutive relationship of concrete, and proposed the relevant constitutive models based on the experimental results. Details of the several commonly used constitutive models of concrete are shown in Table 8, where *σ* and *ε* are the stress and strain of concrete, respectively; *σ*_0_ and *ε*_0_ represent the peak stress and the corresponding strain of concrete, respectively; *σ_cu_* is the ultimate strain of concrete; *E*_0_ is the initial elastic modulus of concrete, and *E_s_* = *σ*_0_/*ε*_0_, is the secant modulus between peak point (*σ*_0_, *ε*_0_) and the origin point.

### 6.2. Model Performance

**Figure 14:**
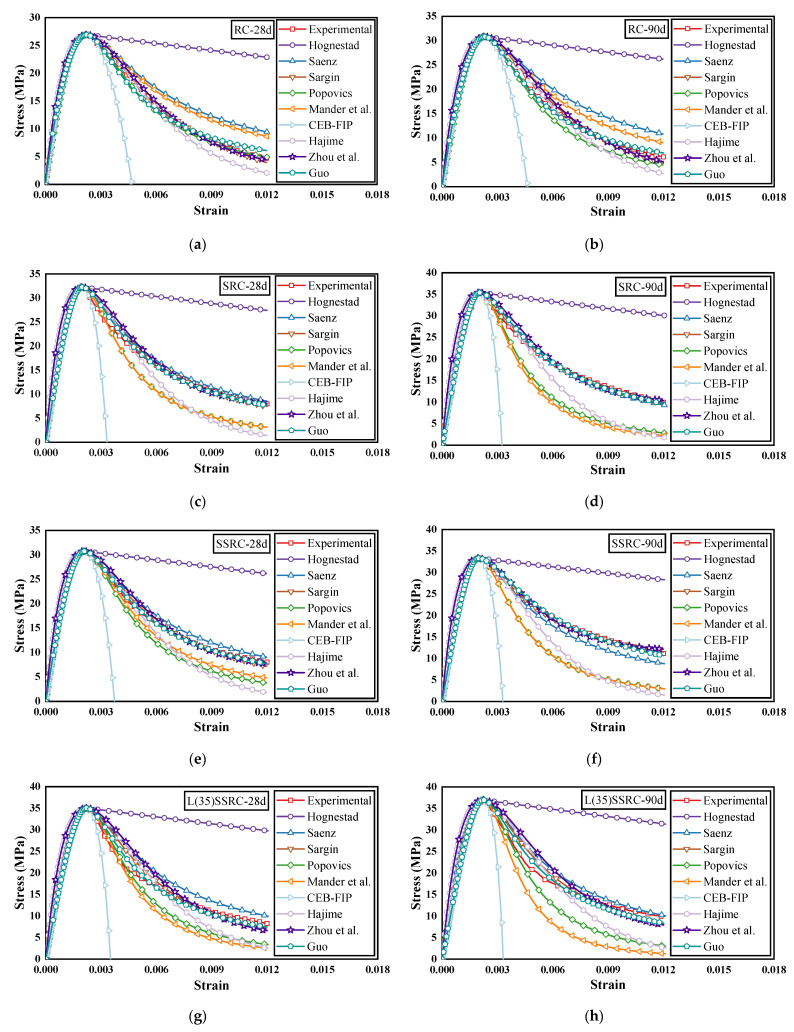

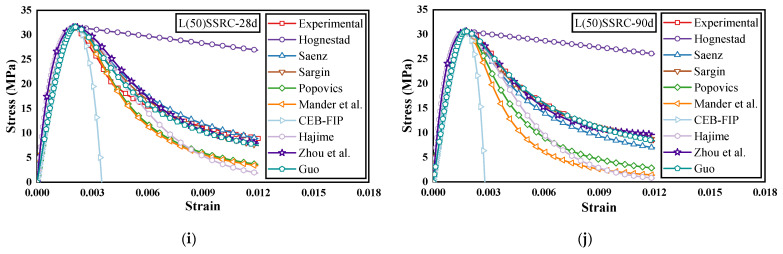
Comparison of existing stress-strain models with experimental results (**a**) RC-28d, (**b**) RC-90d, (**c**) SRC-28d, (**d**) SRC-90d, (**e**) SSRC-28d, (**f**) SSRC-90d, (**g**) L(35)SSRC-28d, (**h**) L(35)SSRC-90d, (**i**) L(50)SSRC-28d and (**j**) L(50)SSRC-90d.

In Sargin [64] model, parameter *D* is used to adjust the softening characteristics of the stress-strain curve. Parameter *C* in Zhou et al. [69] model is used to adjust the shape of the stress-strain curve, and parameters *a* and *b* in Guo [70] model control the ascending and descending sections of the stress-strain curve, respectively. By comparing the model with the experimental results, the optimal values of the parameters in the model and their correlation coefficients *R*^2^ are determined, as shown in Table 9 and Table 10. The comparison between the existing stress-strain analytical models and the experimental results of RC, SRC, SSRC, L(35)SSRC, and L(50)SSRC are demonstrated in Figure 14.

It is remarked that all the models could effectively predict the rising phase of the stress-strain curve. However, neither Hognestad [61,62] model nor CEB-FIP [67] model could effectively predict the descent part of the stress-strain curve, and Hognestad [61,62] model overestimates the descending part of the curve while CEB-FIP [67] model underestimates the descending portion of the curve. For RC, both Saenz [63] model and Mander et al. [66] model overestimate the decline stage of the curve, while Hajime [68] model shows the opposite tendency; although Popovics [65] model predicts a stress-strain curve that is close to the experimental results at the age of 28d, it underestimates the decline part of the curve at the age of 90 days. Similarly, models such as Popovics [65], Mander et al. [66] and Hajime [68] underestimate the descending part of the curves of SRC, SSRC, L(35)SSRC and L(50)SSRC. The stress-strain curve predicted by Saenz [63] model is relatively close to the experimental results, but the effectiveness of the prediction model varies with the ages. As clearly shown in Figure 14, models such as Sargin [64], Zhou et al. [69] and Guo [70] predict the stress-strain curves that are very close to experimental results for all the concrete types, and the prediction model are effective at the age of both 28 and 90 days. In addition, Saenz [63] model can also efficiently predict the stress-strain behavior of SRC.

### 6.3. Constitutive Model of Concrete

**Figure 15:**
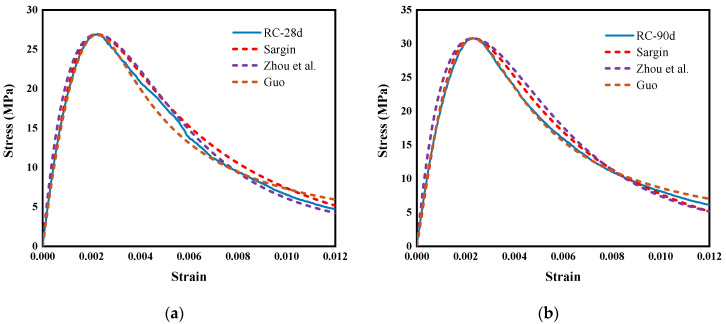

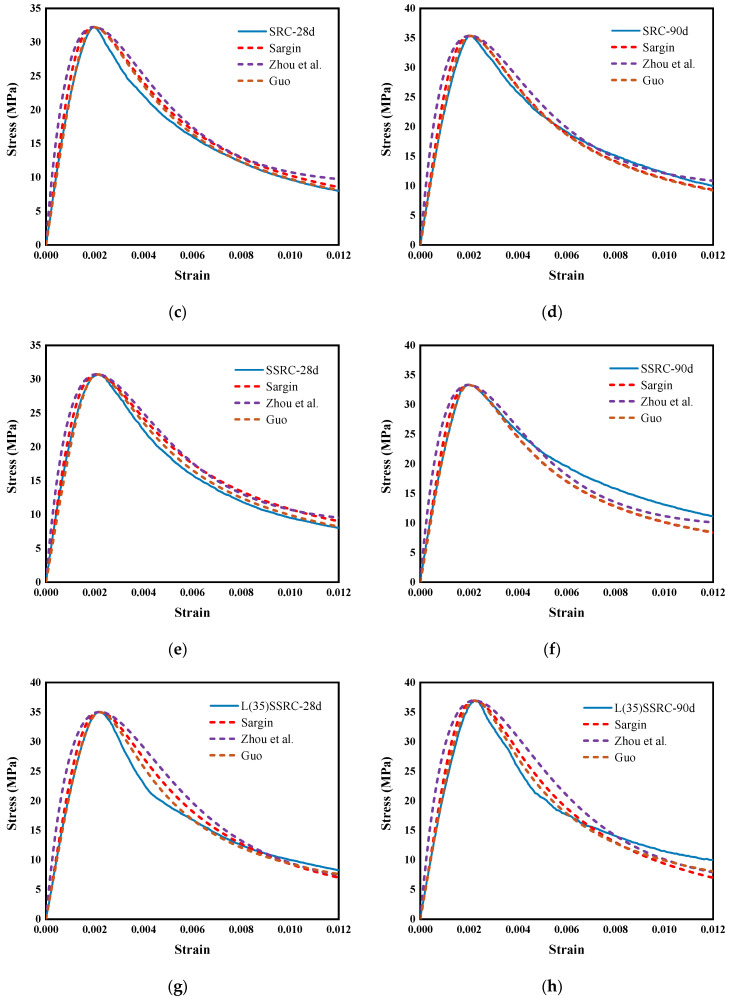

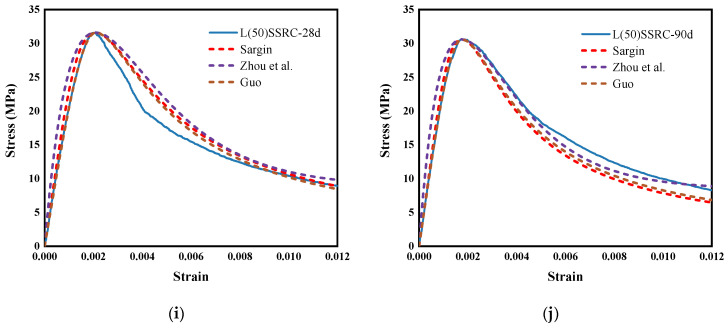
Comparison of proposed models with experimental results (**a**) RC-28d, (**b**) RC-90d, (**c**) SRC-28d, (**d**) SRC-90d, (**e**) SSRC-28d, (**f**) SSRC-90d, (**g**) L(35)SSRC-28d, (**h**) L(35)SSRC-90d, (**i**) L(50)SSRC-28d and (**j**) L(50)SSRC-90d.

In order to establish the constitutive models of RC, SRC, SSRC, L(35)SSRC, and L(50)SSRC, Sargin [64] model, Zhou et al. [69] model and Guo [70] model were further analyzed. A constitutive model that can effectively predict the stress-strain behavior of concrete is established by modifying the optimal values of the parameters in the model. Considering the influence of concrete age on the stress-strain curve, the relevant parameters are recommended, as shown in Table 11. Figure 15 demonstrates the comparison between the proposed model and the experimental results. It is remarked that the stress-strain curve of the proposed analytical model is very close to the experimental ones. It could be concluded that the proposed model can effectively predict the stress-strain relationships of RC, SRC, SSRC, L(35)SSRC and L(50)SSRC.

## 7. Conclusions

The main objective of this study is to explore the mechanical performance of sustainable concrete incorporating LC^3^, SWSS and strengthened recycled coarse aggregate, and the constitutive relationships of various concrete types were established based on the experimental results. The following conclusions are drawn:

(1) The mechanical performance of concrete using SRCA is remarkably improved, and the increasing ratio of 28-day compressive strength attains 19.34%. Slight reduction of compressive strength is observed when SWSS is incorporated, while, the situation is alleviated when 35% LC^3^ is added. The compressive strength of L(35)SSRC is the highest at both 28 and 90 days. Moreover, the failure mode of concrete cylinders is altered with the usage of SRCA. More cracks are generated, and the cylinders keep almost intact without concrete blocks peeling off from the cylinders.
(2) The incorporation of both LC^3^ and SWSS refines the pore structure of concrete matrix. Although the total porosity is increased, the increment is mainly attributed to the rising of the volume of gel pores, which exhibit negligible influence on the strength of concrete.
(3) SWSS leads to the formation of Friedel’s salt and additional ettringite, and the combination of LC^3^ and SWSS further promotes the formation of Friedel’s salt owing to the high content of aluminate phases in calcined clay. The content of Ca(OH)_2_ is reduced due to the pozzolanic reaction with the reactive components in calcined clay, while the content of CaCO_3_ is increased with the incorporation of limestone.
(4) Based on the comparison analyses between the commonly used stress-strain models and the experimental results, the predictive constitutive models of the sustainable concretes are proposed by considering the influence of concrete age.

## Figures and Tables

**Table 1:** Properties of RCA and SRCA.

	Bulk Density (kg/m^3^)	Crushing Index (%)	Water Absorption Ratio (%)
RCA	2617	19.49	6.11
SRCA	2653	12.21	6.02

**Table 2:** Mixture proportions of the strengthening fiber reinforced slurry (kg/m^3^).

Cement	Fly Ash	Water	PE Fiber	Quick-Setting Accelerator	Superplasticizer
1130	380	580	14	30	21

**Table 3:** Properties of PE fiber.

Length (mm)	Diameter (μm)	Aspect Ratio	Strength (GPa)	Elastic Modulus (GPa)	Elongation at Break (%)	Density (kg/m^3^)
12	25	480	2.9	116	2.42	970

**Table 4:** Chemical composition and physical properties of the cementitious materials (%).

Compounds	OPC	Calcined Clay	Limestone
SiO_2_	20.271	53.732	0.309
Al_2_O_3_	5.184	39.405	0.130
K_2_O	0.809	4.229	0.040
Fe_2_O_3_	4.087	2.056	-
MgO	0.086	0.307	0.769
TiO_2_	0.349	0.18	-
SO_3_	2.684	0.087	-
CaO	63.913	0.102	98.715
Rb_2_O	-	0.037	-
SrO	0.030	-	0.037
MnO	0.098	-	-
ZnO	0.079	-	-
P_2_O_5_	0.044	0.037	-
Na_2_O	0.086	-	-
Specific surface area (m^2^/g)	0.96	11.53	0.25
Specific gravity	3.07	2.48	2.70

**Table 5:** Properties of sea sand and desalted sea sand.

Types	Sea Sand	Desalted Sea Sand
Fineness modulus	2.3	2.2
Sludge content (%)	2.4	0.4
Mud mass content (%)	0.6	0.4
Apparent density (kg/m^3^)	2620	2620
Bulk density (kg/m^3^)	1480	1450
Tight density (kg/m^3^)	1670	1680
Shell content (%)	0.4	3.6
Chloride ion content (%)	0.159	0.007
Sulfates and sulfides (%)	0.90	0.81
Organic matter	Qualified	Qualified

**Table 6:** Main ion content in seawater and tap water (unit: mg/L).

Types	Na^+^	K^+^	Ca^2+^	Mg^2+^	F^−^	Cl^−^	${SO}_{4}^{2-}$	${CO}_{3}^{2-}$
Seawater	4.24 × 10^3^	380	390	1.25 × 10^3^	<0.10	1.97 × 10^3^	5.24 × 10^3^	11.78
Tap water	2.69	11.20	6.65	1.76	0.17	10.90	7.19	<0.10

**Table 7:** Concrete mixture proportions (kg/m^3^).

Concrete	OPC	Calcined Clay	Limestone	Tap Water	Desalted Sea Sand	Seawater	Sea Sand	RCA	SRCA	Effective w/b
RC	538	-	-	274	576			1070		0.39
SRC	538	-	-	274	576			-	1070	0.39
SSRC	538	-	-	-	-	274	576	-	1070	0.39
L(35)SSRC	360	119	59	-	-	274	576	-	1070	0.39
L(50)SSRC	296	161	81	-	-	274	576	-	1070	0.39

**Table 8:** Existing constitutive models.

Model	Stress-Strain Model
Hognestad [61,62]	$\mathrm{For}\;\varepsilon \leq \varepsilon_{0}$ $,\;\sigma =\sigma_{0}\left[2\left(\frac{\varepsilon }{\varepsilon_{0}}\right)-{\left(\frac{\varepsilon }{\varepsilon_{0}}\right)}^{2}\right]$
	$\mathrm{For}\;\varepsilon_{0}<\varepsilon \leq \varepsilon_{cu}$ $,\;\sigma =\sigma_{0}\left[1-0.15\left(\frac{\varepsilon -\varepsilon_{0}}{\varepsilon_{cu}-\varepsilon_{0}}\right)\right]$
Saenz [63]	$\sigma =\frac{E_{0}\varepsilon }{1+\left(\frac{E_{0}}{E_{s}}-2\right)\frac{\varepsilon }{\varepsilon_{0}}+{\left(\frac{\varepsilon }{\varepsilon_{0}}\right)}^{2}}$
Sargin [64]	$\sigma =\sigma_{0}\frac{\frac{E_{0}}{E_{s}}\frac{\varepsilon }{\varepsilon_{0}}+\left(D-1\right){\left(\frac{\varepsilon }{\varepsilon_{0}}\right)}^{2}}{1+\left(\frac{E_{0}}{E_{s}}-2\right)\frac{\varepsilon }{\varepsilon_{0}}+D{\left(\frac{\varepsilon }{\varepsilon_{0}}\right)}^{2}}$
Popovics [65]	$\sigma =\sigma_{0}\frac{\varepsilon }{\varepsilon_{0}}\frac{n}{n-1+{\left(\varepsilon /\varepsilon_{0}\right)}^{n}}$
	$n=0.4\times 10^{-3}\sigma_{0}+1.0$$,\;\mathrm{and}\;\sigma_{0}$ is in psi (1 MPa = 145 psi)
Mander et al. [66]	$\sigma =\sigma_{0}\frac{\varepsilon }{\varepsilon_{0}}\frac{n}{n-1+{\left(\varepsilon /\varepsilon_{0}\right)}^{n}}$
	$n=\frac{E_{0}}{E_{0}-E_{s}}$
CEB-FIP [67]	$\frac{\sigma }{\sigma_{0}}=\frac{1.1\frac{E_{0}}{E_{s}}\frac{\varepsilon }{\varepsilon_{0}}-{\left(\frac{\varepsilon }{\varepsilon_{0}}\right)}^{2}}{1+\left(1.1\frac{E_{0}}{E_{s}}-2\right)\frac{\varepsilon }{\varepsilon_{0}}}$
Hajime [68]	$\sigma =6.75\sigma_{0}\left(e^{-0.812\frac{\varepsilon }{\varepsilon_{0}}}-e^{-1.218\frac{\varepsilon }{\varepsilon_{0}}}\right)$
Zhou et al. [69]	$\sigma =\frac{4\sigma_{0}}{{\left(1+C\right)}^{2}}\left[e^{-\frac{\varepsilon }{\varepsilon_{0}}\ln\left(\frac{2}{1-C}\right)}+C\right]\left[1-e^{-\frac{\varepsilon }{\varepsilon_{0}}\ln\left(\frac{2}{1-C}\right)}\right]$
Guo [70]	$\mathrm{For}\;0\leq \frac{\varepsilon }{\varepsilon_{0}}<1$ $,\;\frac{\sigma }{\sigma_{0}}=a\frac{\varepsilon }{\varepsilon_{0}}+\left(3-2a\right){\left(\frac{\varepsilon }{\varepsilon_{0}}\right)}^{2}+\left(a-2\right){\left(\frac{\varepsilon }{\varepsilon_{0}}\right)}^{3}$
	$\mathrm{For}\;\frac{\varepsilon }{\varepsilon_{0}}\geq 1$ $,\;\frac{\sigma }{\sigma_{0}}=\frac{\frac{\varepsilon }{\varepsilon_{0}}}{b{\left(\frac{\varepsilon }{\varepsilon_{0}}-1\right)}^{2}+\frac{\varepsilon }{\varepsilon_{0}}}$

**Table 9:** Comparison of model and experimental results at 28 days.

Model		RC-28d	SRC-28d	SSRC-28d	L(35)SSRC-28d	L(50)SSRC-28d
Sargin	*D*	0.76	0.96	0.93	0.89	0.94
	*R* ^2^	0.99	0.98	0.99	0.95	0.94
Zhou et al.	*C*	0.02	0.06	0.06	0.03	0.06
	*R* ^2^	0.98	0.92	0.95	0.85	0.86
Guo	*a*	2.11	1.45	1.68	1.42	1.45
	*R* ^2^	0.99	0.99	0.99	0.99	0.99
	*b*	0.97	0.75	0.77	1.03	0.82
	*R* ^2^	0.98	0.99	0.99	0.97	0.95

**Table 10:** Comparison of model and experimental results at 90 days.

Model		RC-90d	SRC-90d	SSRC-90d	L(35)SSRC-90d	L(50)SSRC-90d
Sargin	*D*	0.77	1.04	1.12	0.93	1.09
	*R* ^2^	0.98	0.98	0.99	0.95	0.99
Zhou et al.	*C*	0.02	0.08	0.10	0.04	0.09
	*R* ^2^	0.96	0.92	0.94	0.84	0.95
Guo	*a*	1.93	1.33	1.44	1.27	1.38
	*R* ^2^	0.99	0.99	0.99	0.99	0.99
	*b*	1.02	0.66	0.50	0.95	0.54
	*R* ^2^	0.99	0.99	0.99	0.97	0.99

**Table 11:** Proposed model.

Model		RC	SRC	SSRC	L(35)SSRC	L(50)SSRC
Sargin	*D*	0.8	1.0	1.0	0.9	1.0
Zhou et al.	*C*	0.02	0.08	0.08	0.04	0.08
Guo	*a*	2.0	1.4	1.4	1.4	1.4
	*b*	1.0	0.7	0.7	1.0	0.7

## Data Availability

The data presented in this study are available on request from the corresponding author.
